# Enforcing ATP hydrolysis enhanced anaerobic glycolysis and promoted solvent production in *Clostridium acetobutylicum*

**DOI:** 10.1186/s12934-021-01639-7

**Published:** 2021-07-29

**Authors:** Zongjie Dai, Yan Zhu, Hongjun Dong, Chunhua Zhao, Yanping Zhang, Yin Li

**Affiliations:** 1 CAS Key Laboratory of Microbial Physiological and Metabolic Engineering, State Key Laboratory of Microbial Resources, Institute of Microbiology, Chinese Academy of Sciences, 1 Beichen West Road, Chaoyang District, Beijing, 100101 China; 2 CAS Key Laboratory of Systems Microbial Biotechnology, Tianjin Institute of Industrial Biotechnology, Chinese Academy of Sciences, Tianjin, 300308 China; 3 Present Address: Infection and Immunity Program and Department of Microbiology, Biomedicine Discovery Institute, Monash University, Melbourne, VIC 3800 Australia; 4 University of Chinese Academy of Sciences, Beijing, 100049 China

**Keywords:** Anaerobic fermentation, *Clostridium acetobutylicum*, ABE fermentation, ATP hydrolysis, F_1_-ATPase, Acidogenesis, Solventogenesis

## Abstract

**Background:**

The intracellular ATP level is an indicator of cellular energy state and plays a critical role in regulating cellular metabolism. Depletion of intracellular ATP in (facultative) aerobes can enhance glycolysis, thereby promoting end product formation. In the present study, we examined this s trategy in anaerobic ABE (acetone-butanol-ethanol) fermentation using *Clostridium acetobutylicum* DSM 1731.

**Results:**

Following overexpression of *atpAGD* encoding the subunits of water-soluble, ATP-hydrolyzing F_1_-ATPase, the intracellular ATP level of 1731(pITF_1_) was significantly reduced compared to control 1731(pIMP1) over the entire batch fermentation. The glucose uptake was markedly enhanced, achieving a 78.8% increase of volumetric glucose utilization rate during the first 18 h. In addition, an early onset of acid re-assimilation and solventogenesis in concomitant with the decreased intracellular ATP level was evident. Consequently, the total solvent production was significantly improved with remarkable increases in yield (14.5%), titer (9.9%) and productivity (5.3%). Further genome-scale metabolic modeling revealed that many metabolic fluxes in 1731(pITF_1_) were significantly elevated compared to 1731(pIMP1) in acidogenic phase, including those from glycolysis, tricarboxylic cycle, and pyruvate metabolism; this indicates significant metabolic changes in response to intracellular ATP depletion.

**Conclusions:**

In *C. acetobutylicum* DSM 1731, depletion of intracellular ATP significantly increased glycolytic rate, enhanced solvent production, and resulted in a wide range of metabolic changes. Our findings provide a novel strategy for engineering solvent-producing *C. acetobutylicum*, and many other anaerobic microbial cell factories.

**Supplementary Information:**

The online version contains supplementary material available at 10.1186/s12934-021-01639-7.

## Background

The fluctuation of petrol fuel supply and associated environmental issues place critical challenges to global economy, thereby motivating alternative of microbial production of biofuels and chemicals from renewable feedstocks [1, 2]. *Clostridium acetobutylicum* is a model anaerobic bacterium and well-known for industrial production of organic solvents including acetone, butanol and ethanol (i.e. ABE fermentation). ABE fermentation involves typically two physiological phases, acidogenesis and solventogenesis. During the acidogenic phase, the bacterial cells produce energy via glycolysis, acetate and butyrate generation and ATP synthase, whereas during the solventogenic phase, the cells use glycolysis and ATP synthase for solvent production and energy metabolism, meanwhile re-assimilate the extracellular accumulated acids to generate solvents and survive acid stress [3]. Therefore, glycolysis is a major source of energy and critical for ABE fermentation using *C. acetobutylicum*.

Glycolysis is a well-conserved metabolic pathway in nearly all types of organisms, and it is subject to regulation of many factors [4]. Transcriptionally, catabolite control protein A (CcpA) positively regulates the expression of glycolysis genes in *C. acetobutylicum* [5]. At metabolite level, intracellular ATP is a key factor regulating the activities of many rate-limiting enzymes in glycolysis [6], such as hexose kinase, phosphofructokinase and pyruvate kinase. Bacterial F_1_F_o_-ATP synthase uses the transmembrane proton potential to synthesize ATP from ADP and it is a major ATP producer in a cell [7, 8]. The F_1_F_o_-ATP synthase consists of a membrane integrated proton channel F_o_ and a cytoplasmic ATP hydrolase (ATPase) F_1_. Increasing the proportion of free-form F_1_ by reducing the expression of F_o_ or enhancing the expression of F_1_ could promote ATP hydrolyzation, reduce the intracellular ATP level, and consequently induce glycolysis. Previous studies demonstrated that in many aerobic cell factories decreasing intracellular ATP level forced the cells to maintain a higher substrate uptake rate, promoted glycolysis to regenerate NAD^+^, and therefore enhanced fluxes towards end products. Specially, deletion of the F_o_-encoding genes *atpFH* disrupted oxidative phosphorylation and led to a two-fold increase of glycolytic flux in *Escherichia coli* [9]. Overexpression of F_1_ α, β and γ subunits remarkably decreased intracellular ATP/ADP ratio and resulted in 2.7, 3.0, and 1.2-fold increased glycolytic flux in *E. coli*, *Lactococcus lactis* and *Lactobacillus plantarum*, respectively [10–12]. Increasing relative abundance of F_1_ unit also increased the glucose consumption in *Bacillus subtilis* [13] and *Corynebacterium glutamicum* [14]. All these efforts were accomplished in aerobic or facultative aerobic microbes; however, whether this strategy is applicable in obligate anaerobes remains unclear.

In the present study, we significantly decreased the intracellular ATP level of *C. acetobutylicum* DSM 1731 via overexpression of native F_1_-ATPase genes. As a result, we observed a remarkably enhanced glucose utilization as well as a substantially improved solvent production. Genome-scale metabolic modelling was employed to examine the metabolic changes at the network level. Overall, we have demonstrated that the strategy of intracellular ATP depletion can be applied in anaerobic cell factories such as *C. acetobutylicum*, to enhance glycolysis and improve end product formation.

## Results

### Reduced intracellular ATP level and ATP/ADP ratio by overexpression of F_1_-ATPase genes

**Fig. 1:**
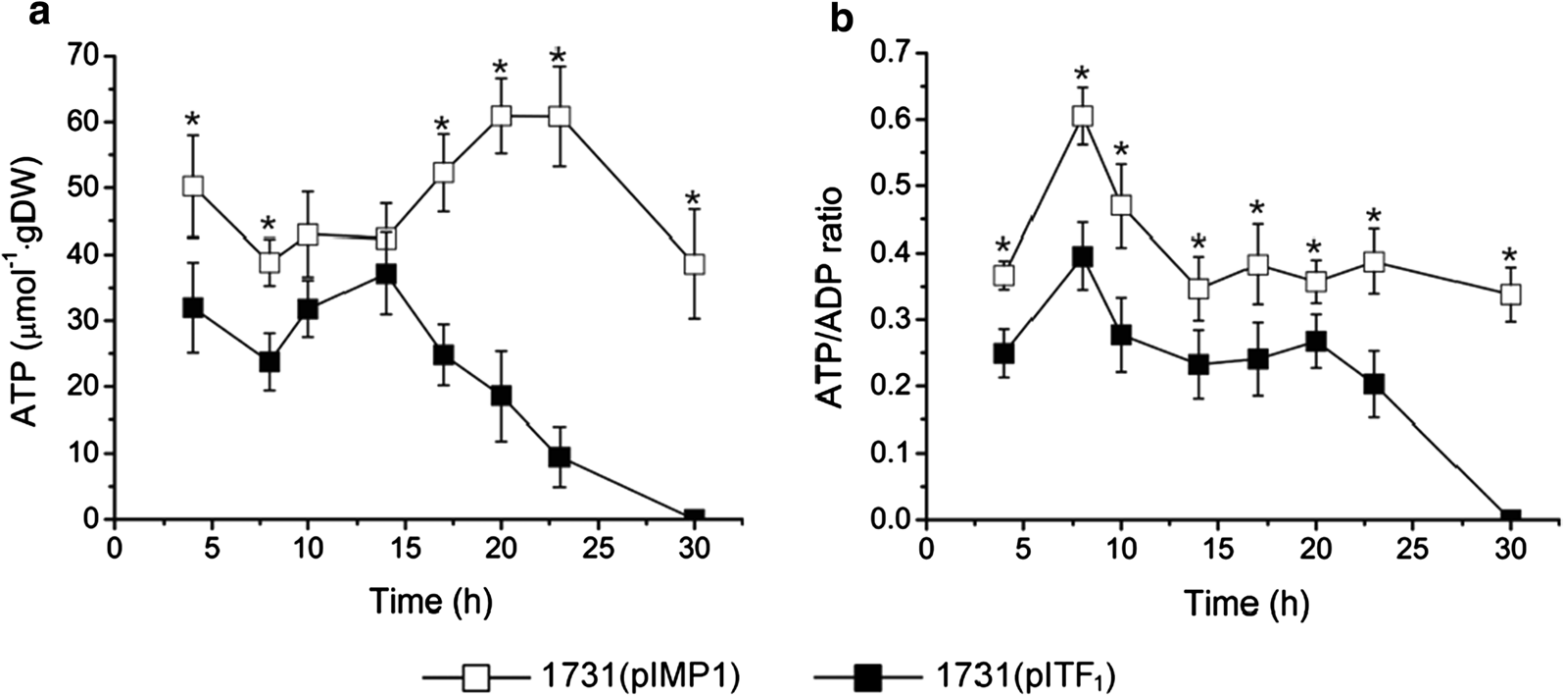
Decreased ATP level (**a**) and ATP/ADP ratio (**b**) in overexpression strain 1731(pITF_1_) compared to vector control 1731(pIMP1) at 0–30 h of anaerobic ABE fermentation. All data are represented as mean ± s.d. (standard deviation, samples were collected from 3 independent bioreactor runs). Student’s *t* test, ^*^p < 0.01

F_1_-ATPase is the water-soluble component of the F_1_F_o_-ATP synthase and catalyzes ATP hydrolysis. It consists of α_3_β_3_γδε subunits (*atpAGDHC*) in *C. acetobutylicum* DSM 1731. Previous studies reported that the combination of α, β and γ showed the strongest ATPase activity [10, 15]. To reduce the intracellular ATP level, the native F_1_-ATPase genes (*atpAGD*) were cloned and expressed in strain DSM 1731 with a constitutive thiolase promoter using plasmid pITF_1_ (Additional file 1: Fig. S1). As expected, the overexpression strain 1731(pITF_1_) displayed a lower ATP level compared to its vector control 1731(pIMP1) during the entire batch fermentation. The ATP level in strain 1731(pITF_1_) peaked (35 µmol gDW^−1^) at 14 h, and rapidly declined to undetectable level thereafter; whereas the ATP level in 1731(pIMP1) peaked (61 µmol gDW^−1^) at 20 h and slowly decreased to 40 μmol gDW^−1^ at 30 h (Fig. 1a). Additionally, a reduced ATP/ADP ratio was observed as well in strain 1731(pITF_1_) compared to vector control throughout the initial 30-h fermentation (Fig. 1b). Together, these results suggest that overexpression of *atpAGD* has potentially increased the intracellular abundance of F_1_-ATPase and enhanced ATP hydrolysis, thereby causing ATP reduction and ADP accumulation.

### Rapid glucose utilization and fast acidogenic growth in strain 1731(pITF_1_)

**Fig. 2:**
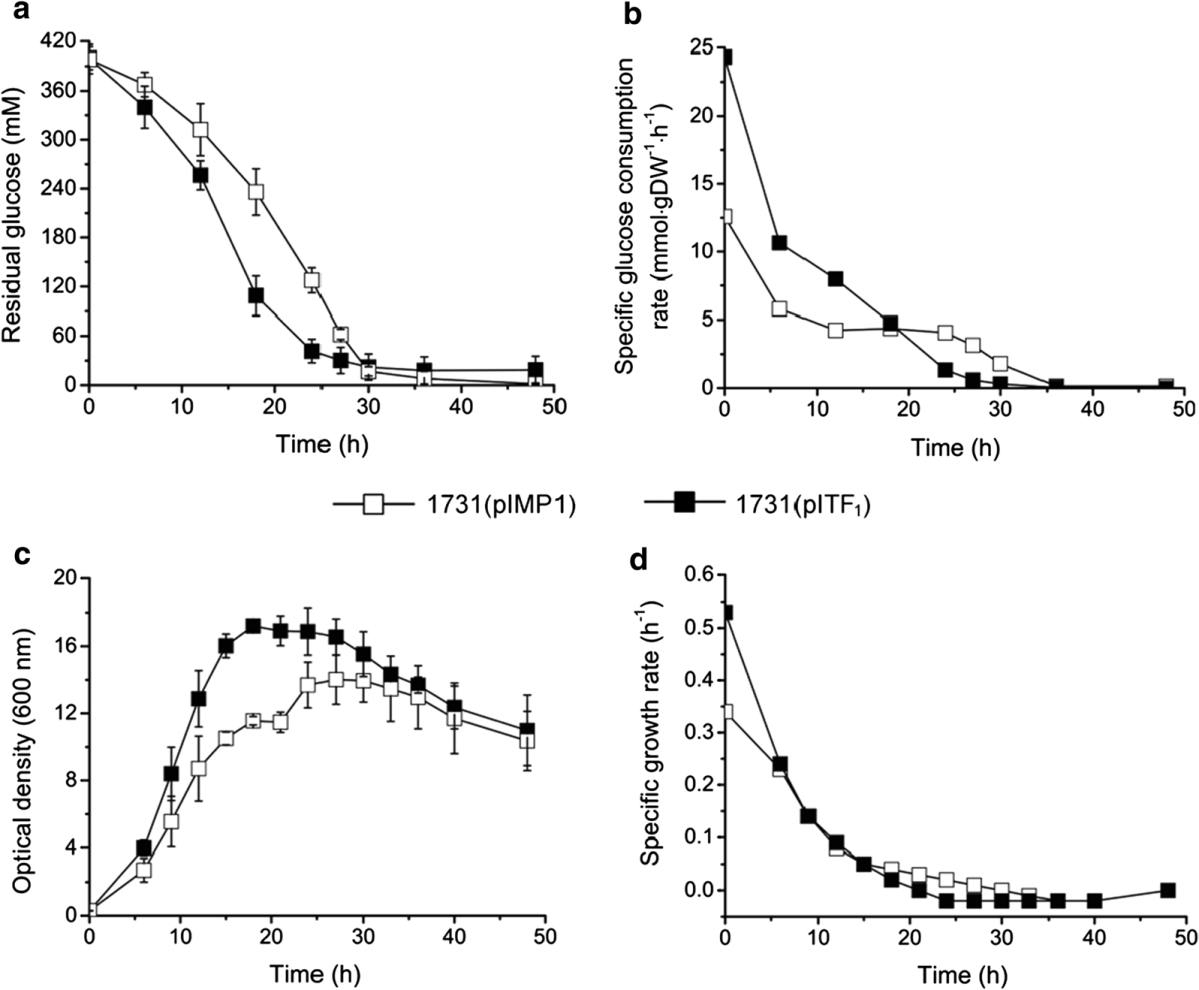
The residual glucose concentration (**a**), specific glucose consumption rate (**b**), optical density (OD_600_) and specific growth rate of 1731(pITF_1_) and 1731(pIMP1) in anaerobic ABE fermentation. The measured glucose concentration (**a**) and optical density (**b**) are shown as mean ± s.d. (samples were collected from 3 independent bioreactor runs)

Overexpression of F_1_-ATPase genes resulted in rapid glucose consumption. Specifically, over the initial 18-h fermentation, 290 mM extracellular glucose was consumed by overexpression strain 1731(pITF_1_); whereas only 162 mM glucose was utilized by vector control strain 1731(pIMP1) (Fig. 2a), representing a 78.8% increase of substrate consumption. A higher volumetric glucose utilization rate (16.15 mmol L^−1^ h^−1^) was achieved by 1731(pITF_1_) compared to vector control (9.03 mmol L^−1^ h^−1^) in the first 18 h. Similarly, the specific glucose utilization rate at 0–12 h was higher in 1731(pITF_1_) than that in 1731(pIMP1) (Fig. 2b, Additional file 2: Table S1). From 18 h onwards, glucose consumption in 1731(pITF_1_) quickly declined to its minimum level, while control strain 1731(pIMP1) still remained a moderate glucose utilization rate till 27 h (Fig. 2b).

Rapid consumption of glucose resulted in fast growth. Specifically, the maximum OD_600_ of 1731(pITF_1_) was 17.2 at 18 h, which is 22.8% higher and 9-h earlier than that of 1731(pIMP1) (14.0 at 27 h) (Fig. 2c). To the best of our knowledge, this is the highest optical density reported thus far for *C. acetobutylicum* in an ABE batch fermentation [16–18]. Moreover, the specific growth rate of 1731(pITF_1_) was substantially higher than that of control at the beginning of fermentation (0 h, 0.52 h^−1^ vs 0.34 h^−1^) but remained similar from 6 h onwards (Fig. 2d, Additional file 2: Table S2).

### Decreased acid accumulation and enhanced solvent production in strain 1731(pITF_1_)

**Fig. 3:**
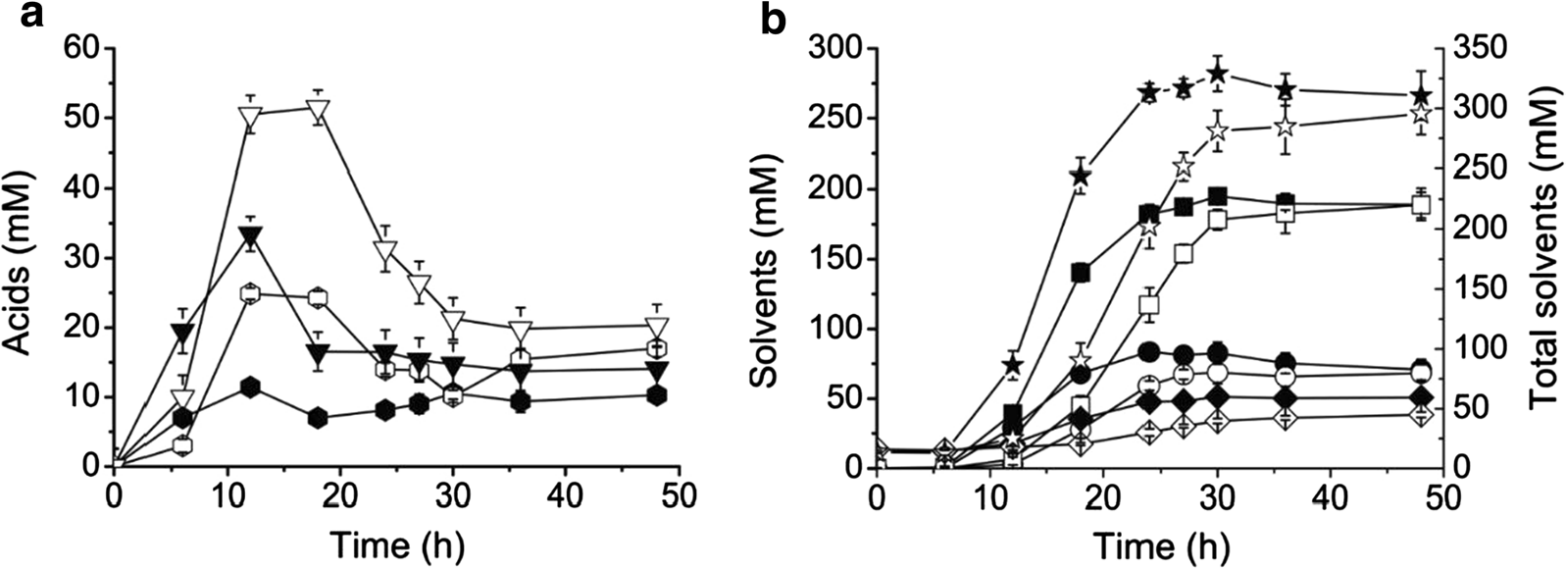
Acid (**a**) and solvent (**b**) profiles in pH-controlled anaerobic ABE fermentations using 1731(pIMP1) (hollow markers) and 1731(pITF_1_) (solid markers). Triangle, butyrate; hexagon, acetate; rectangle, butanol; circle, acetone; diamond, ethanol; asterisk, total solvents. All data are represented as mean ± s.d. (samples were collected from 3 independent bioreactor runs)

ABE fermentation of *C. acetobutylicum* undergoes acidogenesis, subsequent acid re-assimilation and solventogenesis [19]. Compared to the control strain 1731(pIMP1), depletion of intracellular ATP resulted in 33.8% less accumulation of total acids and 9.9% more production of total solvents in 1731(pITF_1_) at 48 h of the batch fermentation (Additional file 1: Table S2). Specifically, along with the fermentation acetate and butyrate achieved the maximum concentrations of 11.4 and 33.5 mM in 1731(pITF_1_) at 12 h, respectively, which were 54.4% and 33.5% less than those in control (Fig 3a, b). At the end of fermentation, the concentrations of acetate and butyrate in 1731(pITF_1_) were 10.3 and 16.7 mM, respectively, whereas the corresponding concentrations were 17.0 and 23.0 mM in 1731(pIMP1). Notably, an earlier re-assimilation of acetate and butyrate is evident in 1731(pITF_1_) compared with 1731(pIMP1) (Fig. 3a).

As to solvent production, butanol, acetone and ethanol achieved their maximum titers of 194.9 mM, 83.6 mM and 51.4 mM, respectively in 1731(pITF1), representing 3.3%, 22.5% and 33.6% increases compared to those in control (Fig. 3a, b, Additional file 1: Table S2). The total solvent yield and productivity were 31.8% and 0.43 g L^−1^ h^−1^ in 1731(pITF_1_), 14.5% and 5.3% higher than those of 1731(pIMP1), respectively (Additional file 1: Table S2). With a mass balance analysis, we discovered that 8.6%, 31.7%, 3.2%, 3.4%, 1.4% and 6.1% of glucose carbon flowed to acetone, butanol, ethanol, butyrate, acetate and biomass in 1731(pIMP1), respectively; whereas in 1731(pITF1), the corresponding percentages are 11.0%, 34.1%, 4.5%, 2.5%, 0.9% and 7.4%, suggesting an enhanced solvent production and biomass formation in overexpression strain (Additional file 2: Table S3).

### Cellular metabolic changes induced by overexpression of F_1_-ATPase genes

**Fig. 4:**
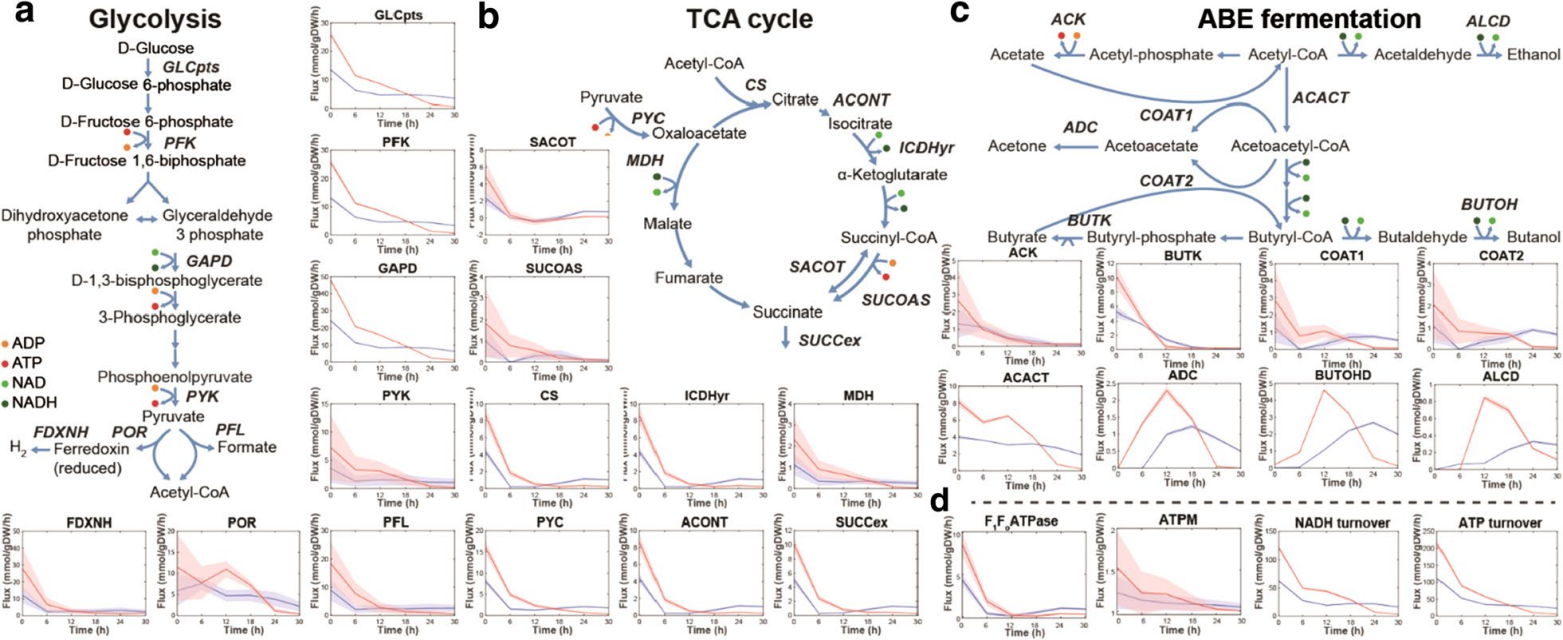
Metabolic flux changes in glycolysis (**a**), TCA cycle (**b**), ABE fermentation (**c**) and energy associated reactions (**d**) of strain 1731(pITF_1_) (red) compared to those of vector control 1731(pIMP1) (blue). Mean and s.d. of 1000 flux samples are shown as line and shading, respectively. *GLCpts* glucose phosphotransferase, *PFK* phosphofructokinase, *GAPD* glyceraldehyde-3-phosphate dehydrogenase, *PYK* pyruvate kinase, *FDXNH* hydrogen ferredoxin oxidoreductase, *POR* pyruvate ferredoxin oxidoreductase, *PFL* pyruvate formate lyase, *CS* citrate synthase, *ACONT* aconitase, *ICDHyr* isocitrate dehydrogenase, *MDH* malate dehydrogenase, *PYC* pyruvate carboxylase, *SACOT* succinyl-CoA:acetoacetate CoA-transferase, *SUCOAS* succinyl-CoA synthetase, *SUCCex* succinate cross-membrane transport, *ACK* acetate kinase, *BUTK* butyrate kinase, *COAT1* acetate-acetoacetate CoA transferase, *COAT2* butyrate-acetoacetate CoA transferase, *ALCD* alcohol/aldehyde dehydrogenase, *ADC* acetoacetate decarboxylase, *ACACT* acetyl-CoA C-acetyltransferase, *BUTOH* butanol dehydrogenase, *ATPM* maintenance ATP hydrolysis

The intracellular ATP allosterically regulates the activity of many metabolic enzymes [6], and its depletion could result in significant metabolic flux changes at network level. A genome-scale metabolic model (*i*Cac20) was constructed for *C. acetobutylicum* DSM 1731 using genome annotation and literature. The model contained 766 genes, 775 metabolites and 1003 reactions, and it was used to compute the metabolic fluxes in strains 1731(pITF_1_) and 1731(pIMP1) during ABE fermentation from 0 to 30 h (Fig. 4, Additional file 2: Table S4).

Compared to 1731(pIMP1), the glycolytic fluxes of 1731(pITF_1_) were enhanced in acidogenic phase, and then substantially declined to low levels from 12 h onwards (Fig. 4a). In glycolysis, phosphofructokinase (PFK) and pyruvate kinase (PYK) are the key enzymes allosterically inhibited by ATP [6]. The decreased intracellular ATP level in 1731(pITF_1_) might result in an alleviation of allosteric inhibition of PFK and PYK, thereby enhancing the activities of these two enzymes and increasing the overall glycolytic activity in acidogenic phase. Simulation results using model *i*Cac20 indicate that 1731(pITF_1_) might have an increased flux through pyruvate ferredoxin oxidoreductase (POR) and an enhanced hydrogen production via hydrogenase (FDXNH) in acidogenic phase (Fig. 4a) compared to vector control. Hydrogen formation requires NADH; the enhanced glycolysis in 1731(pITF_1_) produced abundant NADH, which then likely increased hydrogen formation. Furthermore, the tricarboxylic acid cycle (TCA) fluxes including those through citrate synthase (CS), aconitase (ACONT), isocitrate dehydrogenase (ICDHyr), were ~ 2.0 to 7.4-fold higher in 1731(pITF_1_) than those in vector control at 0–12 h, respectively, but were sharply declined to low levels thereafter (Fig. 4b). Together, the enhanced metabolic activity in glycolysis and TCA in acidogenic phase could produce ample energy and building blocks (e.g. amino acids, nucleotides and lipids) for exponential growth in 1731(pITF_1_) (Fig. 1c).

Within ABE fermentation pathway, the flux through thiolase (ACACT) was 1.3 to 2.1-fold higher in strain 1731(pITF_1_) than that in control at 0–18 h, but was remarkably low at 24 and 30 h. Similarly, in 1731(pITF_1_) the fluxes through solventogenic reactions including acetoacetate decarboxylase (ADC), CoA transferases (COAT1/COAT2), alcohol dehydrogenases (ALCD and BUTOH) were significantly higher than those in 1731(pIMP1) at 0–12 h of fermentation. (Fig. 4c). At 18 h, the fluxes via ADC and BUTOH in 1731(pITF_1_) started to decline, while the corresponding fluxes in 1731(pIMP1) achieved high levels.

In energy generation (Fig. 4d), the ATP synthetic flux via F_1_F_o_-ATPase in 1731(pITF_1_) was 1.9 to 6.0-fold higher than 1731(pIMP1) at 0–6 h, but rapidly decreased at the following timepoints. The average NADH and ATP turnover rates remained at approximately 1.8–2.3 and 1.7–1.9 fold higher in 1731(pITF_1_) compared to 1731(pIMP1), respectively (Fig. 4d), but declined to a low level after 12 h. Notably, in 1731(pITF_1_), the ATP hydrolysis flux (ATPM) was at a 24% higher level at 0 h, but remained at a similar or even lower level at the following time points compared to that in vector control, suggesting an initially enhanced ATP hydrolysis activity induced by overexpression of F_1_-ATPase subunit genes. Overall, our flux balance analysis (FBA) results indicate that overexpression of F_1_-ATPase genes resulted in a significant impact on bacterial metabolism not only ABE fermentation, but also many other pathways including glycolysis, TCA cycle and energy metabolism.

### Early onset of solventogenesis in F_1_-ATPase overexpression strain 1731(pITF_1_)

**Fig. 5:**
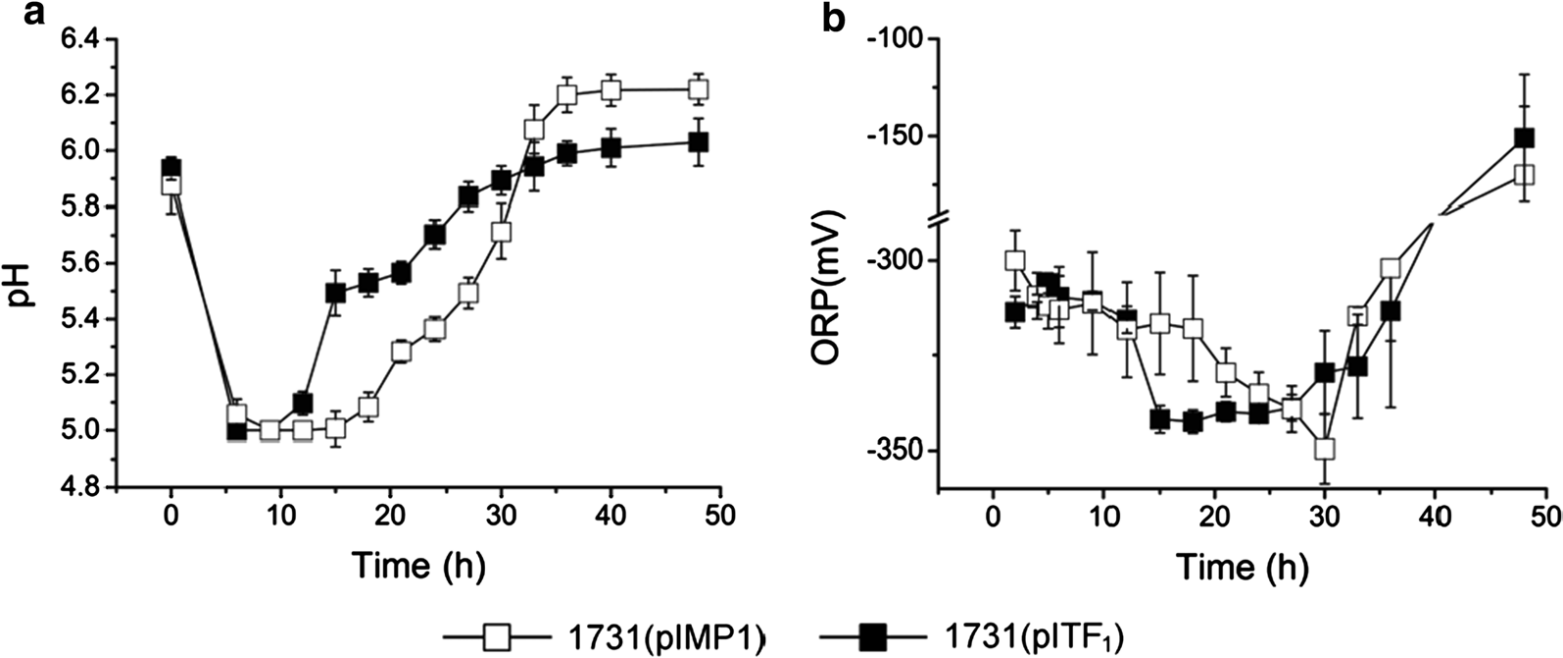
The variations of pH (**a**), ORP (**b**) of 1731(pITF1) and 1731(pIMP1) in anaerobic ABE fermentation. Data of **a** and **b** are shown as mean ± s.d. (samples were collected from 3 independent bioreactor runs)

In ABE fermentation, solventogenesis starts along with re-assimilation of extracellularly accumulated acetate and butyrate, rising extracellular pH, decreasing culture oxidoreductive potential (ORP) [20]. Strain 1731(pITF_1_) started to reutilize acids at 12 h, approximately 6-h earlier than vector control strain 1731(pIMP1) (Fig. 3a). The culture pH of both strains declined to ~ 5.0 at 6 h owing to rapid acid accumulation. Notably, the pH started to rise at 12 h in 1731(pITF_1_) and 18 h in 1731(pIMP1) (Fig. 5a). Together with the advanced reduction of external acetate and butyrate in 1731(pITF_1_) (Fig. 3a), an earlier acid re-assimilation is evident. As an important fermentation parameter, the culture ORP indicates the overall oxidoreductive status of fermentation broth [20], and it rapidly declines when solvents start to accumulate extracellularly. In 1731(pITF_1_), ORP displayed a sharp decrease to its minimum value after 12 h and was remained at approximately − 340 mV till 27 h; whereas in 1731(pIMP1), ORP level slowly decreased to its lowest value at 30 h (Fig. 5b), indicating an earlier solvent accumulation in 1731(pITF_1_). Furthermore, alcohol (i.e. ethanol and butanol) formation is one of the major electron sinks during solventogenesis of *C. acetobutylicum* [21, 22]. The turnover rate of intracellular NADH in 1731(pITF_1_) was much higher than that of control in the first 12 h of fermentation (Fig. 4d, Additional file 2: Table S5); whereas the NADH/NAD ratio in 1731(pITF_1_) remained unchanged (Additional file 1: Fig. S2), implying that a large amount of NADH was rapidly generated via glycolysis to drive alcohol formation without significantly disturbing cellular redox balance. Consistently, at 18 h the ethanol and butanol titers of 1731(pITF_1_) were 2 and 3-fold higher than those in control, respectively (Fig. 3a, 3b). These results together indicate an early commencement of solventogenesis in 1731(pITF_1_) in concomitant with the significantly decreased intracellular ATP level.

## Discussion

The biological production ABE process using anaerobe *C. acetobutylicum* recently attracts extensive interests from both academic and industry owing to the limited supply of petroleum fuel and mounting environmental concerns [23, 24]. Many efforts have been done to improve solvent production, including trimming the competing butyrate biosynthesis pathways [25], overexpressing aldehyde/alcohol dehydrogenase (*adhE1*) [18], and screening solvent tolerant mutants [26]; however, these studies either directly worked on ABE enzymes or purely relied on screening from random mutagenesis. Acid production is one of energy generation pathway of *C. acetobutylicum*; hence, inactivation of butyrate production might impair energy production and result in slow growth and low cell density [25]. Overexpression of aldehyde/alcohol dehydrogenase may result in strong competition of carbon flow with acid biosynthesis, thereby affecting cell growth [18]. Enhancing cellular tolerance to solvent products is an alternative strategy, but it is in concomitant with rapid accumulation of many random mutations potentially with adverse effects [26]; this may be inappropriate for continuous industrial production. Here, we employed a rational strategy of enforcing glycolysis via decreasing intracellular ATP level. To the best of our knowledge, this is the first application of such strategy in ABE fermentation by *C. acetobutylicum*.

Different strategies were applied in aerobic microorganisms to reduce intracellular ATP level and reinforce glycolysis; these include construction of ATP futile cycles by overexpression of pyruvate carboxylase and phosphoenolpyruvate carboxykinase in *Saccharomyces cerevisiae* for ethanol fermentation [27], and overexpression of phosphoenolpyruvate synthase and pyruvate kinase in *E. coli* for lactate production [28]. Notably, both pyruvate carboxylase and pyruvate kinase use pyruvate as a substrate, and ABE fermentation pathway starts with acetyl-CoA from pyruvate cleavage. Therefore, overexpression of these futile cycle enzymes may significantly disturb the flux through pyruvate towards ABE fermentation, thereby unlikely resulting in a high yield of solvents. Whereas overexpression of F_1_-ATPase enforced ATP hydrolysis, without directly competing glycolytic metabolites or diverging glycolytic fluxes to other pathways; it is thus more suitable for metabolic engineering ABE fermentation pathway in *C. acetobutylicum*.

In *C. acetobutylicum* strain DSM 1731, F_1_F_o_-ATP synthase is encoded by operon *atpIBEFHAGDC*. Previous studies has revealed that the membrane-bound proton channel F_o_ is formed by three subunits with the stoichiometry ab_2_c_10–15_, while the soluble F_1_ component consists of subunits α_3_β_3_γδε [7, 8, 15]. F_1_F_o_-ATP synthase catalyzes ATP synthesis using the energy released from proton influx via oxidative phosphorylation; whereas F_1_ component has the secondary function of hydrolyzing cytosolic ATP through the action of a rotational mechanism. Overexpression of F_1_ subunit genes *atpAGD* (Additional file 1: Fig. S1) potentially resulted in accumulation of catalytic F_1_ components and excessive intracellular ATPase activity. As expected, we observed a significantly reduced intracellular ATP level and decreased ATP/ADP ratio in *C. acetobutylicum* (Fig. 1), which is consistent with previous findings in aerobic or facultative microbes [10, 12]. Previous works showed that the maximal specific growth rate was decreased in ATP depleted strains such as *E. coli* [6], *B. subtilis* [13] and *C. glutamicum* [14]. The unexpected increase of specific growth rate before 6 h in 1731(pITF_1_) compared to control may be due to the hydrolyzed ATP was compensated by higher glycolytic (e.g. PYK) and acidogenetic fluxes (PYK, ACK, BUTK) (Fig. 4a, c) which generated ATP in *C. acetobutylicum*.

The traditional batch ABE fermentation suffers from low cell density and low reactor productivity, this could be due to the rapid accumulation of toxic byproducts (e.g. acetate, butyrate, acetone, ethanol and butanol) and/or the insufficient energy supply for bacterial growth [23]. Anaerobic *C. acetobutylicum* uses glycolysis, acid fermentation and membrane-bound F_1_F_o_-ATP synthase to produce ATP. In the present study, the significantly reduced ATP level resulted in substantially increased glucose consumption, fast growth (0–20 h, Fig. 2), and reduced acetate and butyrate accumulation (Fig. 3) compared to vector control. Consequently, strain 1731(pITF_1_) had a 22.8% increase at maximal cell density compared to vector control 1731(pIMP_1_) and a significantly improved solvent volumetric productivity which is necessary to develop effective continuous butanol production [29].

Clostridial ABE fermentation involves two physiological stages, namely acidogenesis and solventogenesis. In acidogenic phase, cells grow exponentially with the production of acetate and butyrate, thereby causing a dramatic decline of extracellular pH. Previous studies revealed that many factors including low pH, large amount of undissolved organic acids and significantly altered energy charge (ATP/ADP ratio) could induce the expression of solventogenic genes (e.g. *adc*, *ctfA/B* and *adhE*) [30, 31]. In the present study, strain 1731(pITF_1_) started acid-assimilation and solventogenesis 6-h earlier than vector control. In addition, with metabolic modelling, we identified many elevated metabolic fluxes during acidogenesis and early induction of solventogenic fluxes in 1731(pITF_1_) (Fig. 4). Overall, the performance of the entire ABE fermentation using 1731(pITF_1_) was significantly improved with better cell growth and higher solvents titer, yield, productivity. High cost is a main limiting factor of commercializing ABE fermentation. The substrate consumption and solvent extraction account for > 30% of total fermentation cost [32]. The higher yield, titer and productivity developed here could significantly reduce the total cost of ABE fermentation. This work suggests that glycolysis via reducing intracellular ATP level is an effective approach to improve solvent production in clostridial cell factory.

## Conclusions

By overexpression of F_1_-ATPase, we significantly reduced the intracellular ATP level in *C. acetobutylicum* industrial strain DSM 1731. The overexpression strain 1731(pITF_1_) exhibited higher cell density, enhanced glycolytic rate, early onset of solventogenesis, significant changes of many metabolic fluxes, and importantly, much improved solvent production than its vector control. Our study, for the first time, demonstrates that the glycolytic rate can be manipulated via altering ATP level in *C. aceotbutylicum*. This strategy can also be employed for metabolic engineering of other anaerobic fermentations.

## Methods

### Bacterial strains and culture conditions

*C. acetobutylicum* strain DSM 1731 and its derivatives (Additional file 1: Table S1) were grown in Reinforced Clostridial Medium (RCM) anaerobically at 37 °C [33, 34]. All *E. coli* strains were grown aerobically in Luria–Bertani (LB) broth at 37 °C with vigorous shaking at 220 rpm [35]. *E. coli* JM109 was employed for gene cloning. Shuttle vector was methylated by transforming *E. coli* ER2275 bearing plasmid pAN1. Ampicillin (100 μg mL^−1^) and erythromycin (50 μg mL^−1^) were used for screening and maintaining of plasmids whenever necessary. Cell growth was monitored by measuring the optical density at 600 nm (OD_600_) with a UV/Vis 2802PC spectrophotometer (Unico, New Jersey, USA).

### Overexpression of F_1_-ATPase in DSM 1731

The F_1_ component genes, *atpAGD* (SMB_G2901-SMB_G2903), and thiolase promoter (P_thl_) were amplified from DSM1731 genome using primers atpAGD-1: 5′-CGCGGATCCATGAACATAAAACCTGAAGAGATAACTTCA-3′, atpAGD-2: 5′-CCGGAATTCTTAGCTTTCCATCATTTTTTTAGCTTT-3′, thl-1: 5′-CGCGTCGACTATATTGATAAAAATAATAATAGTG and thl-2: 3′-CGCGGATCCTTCTTTCATTCTAACTAACCTCCTA. The fragments were then jointed and cloned into the shuttle vector pIMP1 [36]. The derived plasmid was designated as pITF_1._ Insertion of DNA sequence was confirmed by sequencing. Plasmids pIMP1 and pITF_1_ (Additional file 1: Table S1) were methylated before transformation of DSM1731 [37]. Electroporation to DSM1731 and SDS-PAGE for detecting protein overexpression were performed based on previous work [38].

### Batch fermentation

Three independent batch fermentations were performed in two 7.5-L BioFlo 110 fermentors (New Brunswick Scientific, Edison, NJ, USA) containing 3.0 L clostridium growth medium (CGM) [22, 39]. Anaerobic condition was assured by continuously sparging nitrogen into reactor. Refined corn oil (2.7 mL L^−1^) was added for defoaming purpose. The reactor medium (initial pH 7.0) was inoculated with 300-mL preculture of early log-phase (OD_600_ ~ 2.0). Broth pH was monitored by pH meter (Mettler-Toledo) and controlled above 5.0 by supplementation with 6 M ammonia automatically. Broth oxidoreductive potential (ORP) was measured by real-time ORP electrode (Mettler-Toledo). Extracellular metabolites from three independent bioreactors including glucose, butanol, ethanol, acetone, butyrate and acetate were determined by high performance liquid chromatography (HPLC, Agilent Technologies, Santa Clara, CA, USA) [38, 40].

### Quantification of intracellular ATP, ADP, NADH and NAD^+^

Intracellular ATP and ADP were extracted with perchloric acid method as described previously [41] with minor modifications. Samples were collected from three independent bioreactors at multiple time points and subjected to centrifugation at 10,000×*g* for 1 min. The cell pellets were homogenized immediately in 200 μL ice-cold 7% perchloric acid and incubated on ice for 10 min. After centrifugation at 15,000×*g* for 5 min, the supernatant was collected and neutralized using 50 μL 3 M potassium hydroxide, 100 μL 0.4 M Tris and 50 μL 3 M potassium chloride. The mixture was vortexed thoroughly and subjected to another centrifugation at 15,000×*g* for 5 min to remove residual precipitate. The supernatant was then transferred to another sterile centrifuge tube for HPLC analysis. An ion-pair reversed-phase HPLC method was used to determine the intracellular adenine nucleotides level according to previous work [42, 43]. Agilent 1200 HPLC was used with 10 μL injection each time. The mobile phase containing potassium phosphate dibasic (107.5 mM), tetrabutylammonium hydrogen sulphate (2.3 mM) and acetonitrile (6%) at pH 6.25 was pumped through a C18 column (SB-AQ 4.6 × 250 mm, 5-micron, Agilent) at a flow rate of 1 mL min^−1^.

The extraction and determination of intracellular NADH and NAD^+^ were conducted as preciously described [44] with slight modifications. To prepare NADH, 400 μL ice-cold 0.4 M potassium hydroxide was firstly added into 1 mL fermentation broth. The mixture was then incubated at 30 °C for 10 min and subject to a centrifugation at 15,000 × *g* for 10 min at 4 °C. Then, 200 μL supernatant was collected and neutralized to pH 7.5–8.0 by adding 0.4 M hydrochloride acid. For NAD^+^, 400 μL ice-cold 0.4 M hydrochloride acid was added to 1 mL broth. The mixture was incubated at 50 °C for 10 min and centrifuged at 15,000 × *g* for 10 min at 4 °C. Then, 200 μL supernatant was collected and neutralized to pH 7.2–7.4 using 0.4 M potassium hydroxide. The neutralized samples were immediately used for NADH and NAD^+^ determination. The spectrometric enzymatic cycling assay was applied with slight modifications. The assay mixture contained 2 mL buffer (0.15 M glycylglycine/nicotinic acid buffer, pH 7.4), 400 μL phenanziniummethylsulfate (PES) (4 mg mL^−1^), 400 μL thiazolyl-blue (MTT) (5 mg mL^−1^), 70 μL ethanol, and 40 μL alcohol dehydrogenase (60 U mL^−1^). After addition of 50 μL neutralized sample into reaction buffer followed by a brief vortexing, absorption was examined using spectrophotometer for 10 min at 570 nm. Mass balance was calculated by summing up carbon amounts of metabolite products and biomass, and compared with glucose consumption. Specific carbon partition was calculated by normalizing the carbon molar amount of a produced metabolite with the carbon molar amount of the consumed glucose. For biomass, its molar carbon per gram dry weight (36.8 mM C gDW^−1^) was calculated by FBA (see below) with glucose uptake rate set as − 10 mmol⋅gDW^−1^⋅h^−1^.

### GSMM construction for *C. acetobutylicum* DSM 1731

The model construction started with collecting genome annotation (GCA_000218855.1) from GenBank and obtaining metabolic reactions from KEGG (Kyoto Encyclopedia of Genes and Genomes) [45] and BioCyc databases [46]. A draft model was constructed based on the predicted metabolic reactions in KEGG, and then supplemented with the missing metabolites, reactions and genes according to the genome annotation of DSM 1731 and BioCyc pathways. Extensive manual curation was conducted to improve the model, including (i) adding extracellular metabolites, (ii) adding exchange and transport reactions, (ii) filling pathway gaps, and (iii) checking the mass and charge balance of each reaction. The resulting model was compiled in Systems Biology Markup Language [47]. The biomass formation equation consisting of necessary building blocks for growth was created using the one from existing model for ATCC 824 model owing to its very close phylogeny relationship to this type strain [48]. The minimum of non-growth associated maintenance (NGAM) was set to 1 mmol gDW h^−1^, 2.5% of growth associated maintenance (40 mmol gDW h^−1^) according to previous modeling effort [49].

### Constraint-based metabolic modeling

Our FBA is based on an assumption of pseudo steady state where the intracellular metabolite concentrations are invariant but the extracellular metabolite concentrates are variable; the exchange fluxes of extracellular metabolites were estimated from the batch fermentations and used as the constraints of FBA. Specifically, the OD_600_ and external metabolite molar concentrations were employed to estimate the specific rates. First, a shape-preserving piecewise cubic Hermite function from shape language modelling (SLM) MATLAB toolbox (https://au.mathworks.com/matlabcentral/fileexchange/24443-slm-shape-language-modeling) was used for data fitting of OD_600_ values and metabolite molar concentrations, followed by a numerical differentiation to compute the changing rates (i.e. dOD_600_/d*t*, d*c*_glc_/d*t* and d*c*_product_/d*t*). Then the specific growth rates (*µ*), specific glucose consumption rates (*r*_glc_), and specific product secretion rates (*r*_product_) were calculated using equations

1 $$\mu =\frac{d{OD}_{600}}{dt\bullet {OD}_{600}},$$

2 $$r=\frac{dc}{dt\bullet DW},$$

where cell dry weight *DW* is calculated by 0.34 × OD_600_ as previously described [50, 51] (Additional file 1: Fig. S3). The calculated specific rates were employed to constrain the corresponding exchange fluxes in *i*Cac20 with up to 10% variations. The optimal specific growth rate (µ*) was firstly calculated using FBA, and then the solution space was sampled with 1000 points using artificially centered hit-and-run (ACHR) algorithm [52] with the specific growth rate set to ≥ 99% of µ*. The obtained feasible solutions were adjusted by applying loopless FBA (ll-FBA) method [53] to avoid unnecessary flux loops. Student’s *t*-test was conducted to determine the differentially changed metabolic fluxes with FDR (false discovery rate) adjusted *P* < 0.05. Metabolite turnover rates were calculated by summing up all the incoming or outgoing fluxes of the metabolite *i* [54] using equation $\varphi ={\sum }_{j}{S}_{ij}{v}_{j}$.

## Supplementary Information

**Additional file 1**:** Fig. S1**. Plasmid construction and overexpression of *atpAGD* genes in strain DSM1731. **Fig. S2**. NADH/NAD^+^ ratio of 1731(pIMP1) and 1731(pITF_1_) in anaerobic ABE fermentation. **Fig S3**. OD_600_ and the calculated specific growth rates of 1731(pIMP1) and 1731(pITF_1_). **Table S1**. Strains and plasmids used in this study. **Table S2**. Comparison of fermentation results after 48 h fermentation between engineered *C. acetobutylicum *strain and control strain.

**Additional file 2:** Table S1. Specific metabolite consumption or production rate obtained by data fitting. Table S2. Specific growth rates obtained by data fitting. Table S3. Calculation of mass balance. Table S4. Average metabolic fluxes of 1731(pITF1) and 1731(pIMP1) in 0–30 h fermentation. Table S5. Mean and SD values of the computed NADH turnover rates for 1731(pIMP1) and 1731(pITF1) at 0, 6, 12, 18, 24 and 30 h.

## Data Availability

All data in the present study are included in the main text and Additional files.

## Abbreviations

ABE: Acetone-butanol-ethanol
CGM: Clostridial growth medium
RCM: Reinforced clostridial medium
HPLC: High-performance liquid chromatography
GC–MS: Gas chromatography–mass spectrometry
RI: Refractive index
TE: Tris-ethylenediaminetetraacetic acid
SDS-PAGE: Sodium dodecyl sulfate polyacrylamide gel electrophoresis
ORP: Oxidoreductive potential
PES: Phenazinium methylsulfate
MTT: Thiazolyl-blue
NGAM: Non-growth associated maintenance
ACHR: Artificially centered hit-and-run
GSMM: Genome-scale metabolic modeling
