# Supplementary material for: Enforcing ATP hydrolysis enhanced anaerobic glycolysis and promoted solvent production in Clostridium acetobutylicum
Source: Microb Cell Fact. 2021 Jul 29;20:149. doi: 10.1186/s12934-021-01639-7 (PMC8320212; doi:10.1186/s12934-021-01639-7)
Supplement: Supplementary file 1 — Additional file 1: Fig. S1. Plasmid construction and overexpression of atpAGD genes in strain DSM1731. Fig. S2. NADH/NAD+ ratio of 1731(pIMP1) and 1731(pITF1) in anaerobic ABE fermentation. Fig S3. OD600 and the calculated specific growth rates of 1731(pIMP1) and 1731(pITF1). Table S1. Strains and plasmids used in this study. Table S2. Comparison of fermentation results after 48 h fermentation between engineered C. acetobutylicum strain and control strain. [file 12934_2021_1639_MOESM1_ESM.docx]

Additional file 1

**Enforcing ATP hydrolysis enhanced anaerobic glycolysis and promoted solvent production in *Clostridium acetobutylicum***

Zongjie Dai^1,2ⱡ^, Yan Zhu^1,3ⱡ^, Hongjun Dong^1,2^, Chunhua Zhao^1,4^, Yanping Zhang^1*^, Yin Li^1^

^1^CAS Key Laboratory of Microbial Physiological and Metabolic Engineering, State Key Laboratory of Microbial Resources, Institute of Microbiology, Chinese Academy of Sciences, Beijing 100101, China;

^2^CAS Key Laboratory of Systems Microbial Biotechnology, Tianjin Institute of Industrial Biotechnology, Chinese Academy of Sciences, Tianjin 300308, China;

^3^Present address: Infection & Immunity Program and Department of Microbiology, Biomedicine Discovery Institute, Monash University, Melbourne, VIC 3800, Australia;

^4^University of Chinese Academy of Sciences, Beijing 100049, China.

^ⱡ^Equal contribution.

^*^Correspondence addressed to Dr. Yanping Zhang, Institute of Microbiology, Chinese Academy of Sciences, 1 Beichen West Road, Chaoyang District, Beijing, 100101, China; Telephone/Fax: +86 10 64807351; Email: [zhangyp@im.ac.cn](mailto:zhangyp@im.ac.cn)


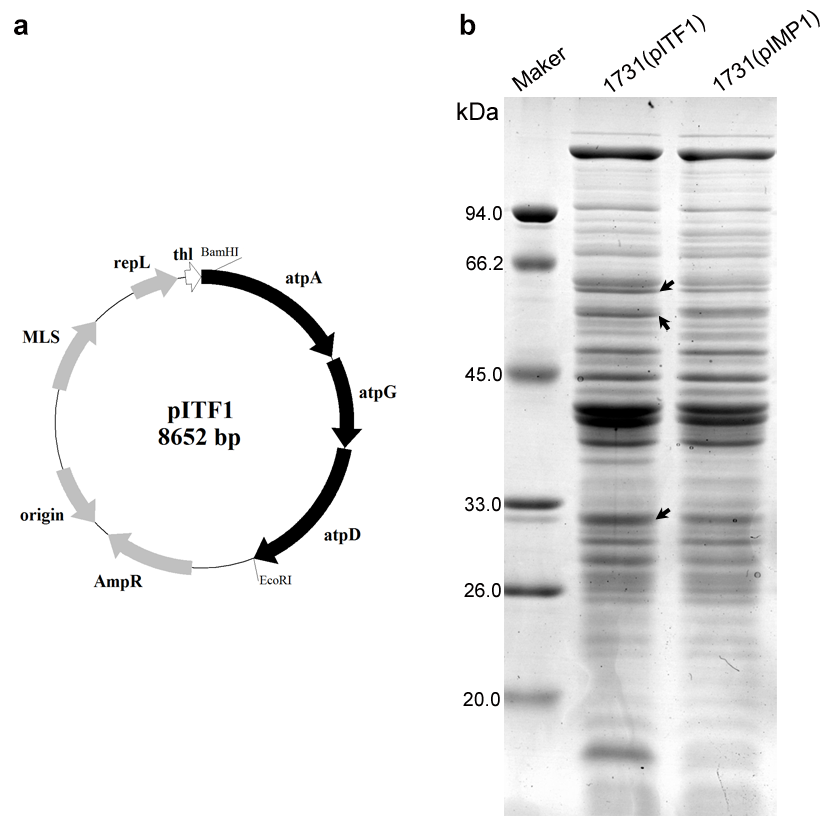
**Fig. S1.** **Plasmid construction and overexpression of *atpAGD* genes in strain DSM1731.** (**a**) Multiple copy plasmid to expressing native *atpAGD* genes with thiolase promoter (thl). (**b**) SDS-PAGE analysis of overexpressing *atpAGD* genes. The overexpressed three proteins (atpA, atpD, atpG) were indicated by the arrow on the right from up to down. Strain DSM1731(pIMP1) was the control strain containing empty vector.


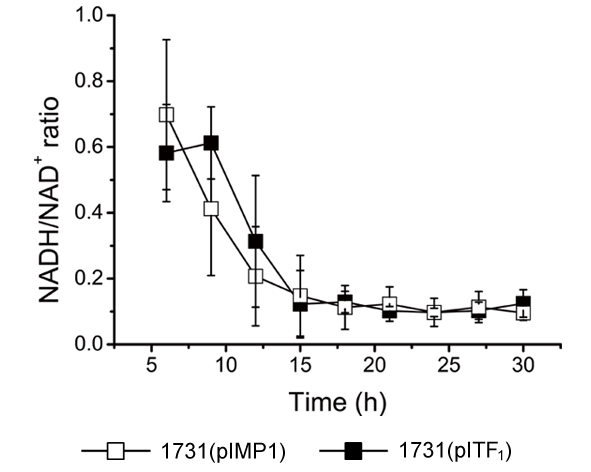


**Fig. S2.** **NADH/NAD^+^ ratio of 1731(pIMP1) and 1731(pITF_1_) in anaerobic ABE fermentation.** Data are shown in mean ± s.d. (*n* = 3).


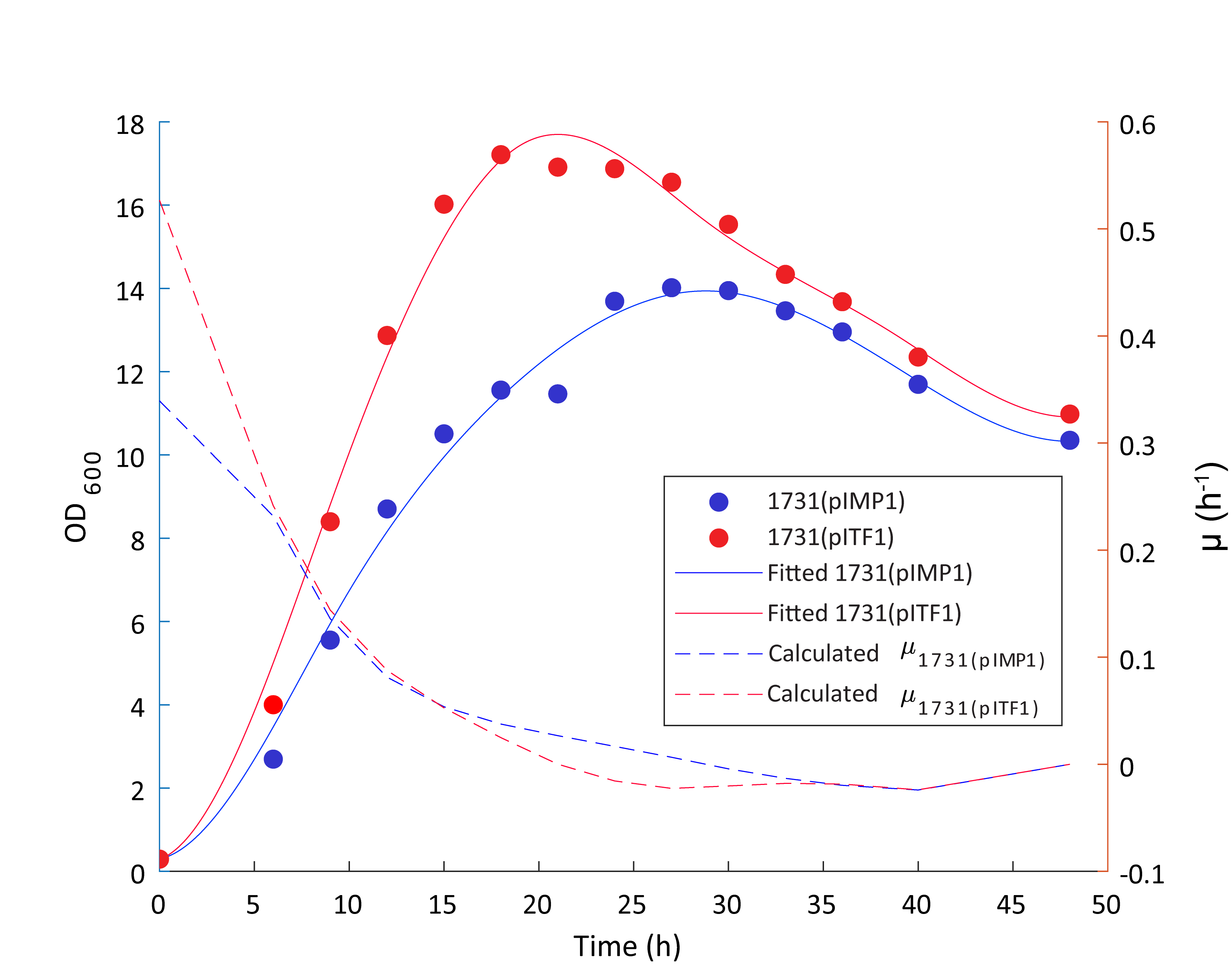


**Fig S3**. **OD_600_ and the calculated specific growth rates of 1731(pIMP1) and 1731(pITF_1_).**

Table S1. Strains and plasmids used in this study

| **Strains and plasmids** |  | **Features** | **Source or reference** |
| --- | --- | --- | --- |
| Strains |  |  |  |
| *C. acetobutylicum* |  |  |  |
|  | DSM1731 | Wide type, the parent strain used in this study | DSMZ |
|  | 1731(pIMP1) | Control strain harboring vector backbone pIMP1 | This study |
|  | 1731(pITF_1_) | *atpADG* overexpressing strain, harboring pITF_1_ | This study |
| *E. coli* |  |  |  |
|  | JM109 | *recA1 mcrB^+^ hsdR17* | Lab storage |
|  | ER2275(pAN1) | used for methylation of plasmid DNA prior to  transformation *C. acetobutylicum* | (Mermelstein et al., 1992) |
| Plasmids |  |  |  |
|  | pAN1 | *ɸ3tI, p15a ori*, Cm^r^ | (Mermelstein et al., 1992) |
|  | pIMP1 | MLS^r^, Amp^r^, shuttle vector of *E. coli*-*C. acetobutylicum* | (Mermelstein et al., 1992) |
|  | pITF | MLS^r^, Amp^r^, pIMP1 derivative for *fdh* expression under *thl* promoter | (Dong et al., 2010) |
|  | pITF_1_ | Used for *atpAGD* expression with *thl*  promoter | This study |

Abbreviations: Amp^r^, ampicillin resistance; Cm^r^, chloramphenicol resistance; MLS^r^, macrolide, lincosamide, and streptogramin B resistance; *ɸ3tI*, methyltransferase gene of *Bacillus subtilis* phage *ɸ3t*I; *thl*, the promoter of thiolase gene in *C. acetobutylicum*; DSMZ, German Collection of Microorganisms and Cell Cultures, Braunschweig, Germany.

Dong, H. J., Zhang, Y. P., Dai, Z. J., Li, Y., 2010. Engineering *Clostridium* strain to accept unmethylated DNA. PLoS One. 5**,** p. e9038.

Mermelstein, L. D., Welker, N. E., Bennett, G. N., Papoutsakis, E. T., 1992. Expression of cloned homologous fermentative genes in *Clostridium acetobutylicum* ATCC 824. Nat. Biotechnol. 10**,** pp. 190-195.

Table S2. Comparison of fermentation results after 48 h fermentation between engineered *C. acetobutylicum* strain and control strain.

|  | Strains | | | |  |  |
| --- | --- | --- | --- | --- | --- | --- |
| Fermentation  characteristics | | 1731(pITF_1_) | 1731(pIMP1) | | |  |
| Number of experiments | | 3 | | 3 | | |
| Glucose (*mM*) | | 381.38 ± 2.79 | | 396.75 ± 16.56 | | |
| Max OD_600_ | | 17.21 ± 0.31 | | 14.02 ± 1.47 | | |
| Acetone (*mM*) | | 83.58 ± 3.62 | | 68.23 ± 6.18 | | |
| Butanol (*mM*) | | 194.98 ± 5.42 | | 188.79 ± 9.26 | | |
| Ethanol (*mM*) | | 51.42 ± 1.54 | | 38.48 ± 1.93 | | |
| Butyrate (*mM*) | |  | |  | | |
| peak | | 33.46 ± 2.51 | | 51.51 ± 2.51 | | |
| final | | 14.07 ± 3.10 | | 20.34 ± 2.99 | | |
| Acetate (*mM*) | |  | |  | | |
| peak | | 11.41 ± 0.82 | | 24.88 ± 1.37 | | |
| final | | 10.27 ± 1.16 | | 16.99 ± 1.15 | | |
| Solvent productivity (g·L^-1^·h^-1^) | | 0.43 | | 0.41 | | |
| Solvent yield (g·g^-1^)  Butanol yield (g·g^-1^) | | 31.8%  21% | | 27.8%  19.6% | | |

*Note:* Means ± SD.
